# 12-Nitro­methyl-14-deoxy­andro­graph­olide

**DOI:** 10.1107/S1600536808018746

**Published:** 2008-06-28

**Authors:** Ji-Cai Quan, Jing Chen, Hao Xu, Chen-Hui Hu, Jin-Tang Wang

**Affiliations:** a College of Science, Nanjing University of Technology, Xinmofan Road No. 5, Nanjing 210009, People’s Republic of China

## Abstract

In the mol­ecule of the title compound {systematic name: 3-[2-(6-hydr­oxy-5-hydroxy­methyl-5,8a-dimethyl-2-methyl­ene­per­hydro-1-napth­yl)-1-(nitro­meth­yl)eth­yl]-2(4*H*)-furan­one}, C_21_H_31_NO_6_, the cyclo­hexane rings have chair conformations. Intra­molecular O—H⋯O hydrogen bonding results in the formation of a six-membered non-planar ring with a twist conformation. In the crystal structure, inter­molecular O—H⋯O hydrogen bonds link the mol­ecules into infinite chains along the *c* axis.

## Related literature

For bond-length data, see: Allen *et al.* (1987 ▶). For ring puckering parameters, see: Cremer & Pople (1975 ▶).
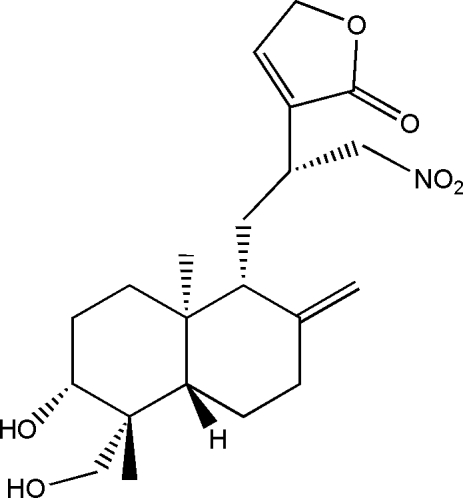


## Experimental

### 

#### Crystal data

C_21_H_31_NO_6_
*M* *_r_* = 393.47
Orthorhombic, 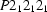

*a* = 11.503 (2) Å
*b* = 13.151 (3) Å
*c* = 13.434 (3) Å
*V* = 2032.2 (7) Å^3^
*Z* = 4
Mo *K*α radiation
μ = 0.09 mm^−1^
*T* = 294 (2) K
0.40 × 0.20 × 0.20 mm

#### Data collection

Enraf–Nonius CAD-4 diffractometer
Absorption correction: ψ scan (North *et al.*, 1968 ▶) *T*_min_ = 0.964, *T*_max_ = 0.982
3993 measured reflections
3643 independent reflections
2711 reflections with *I* > 2σ(*I*)
*R*_int_ = 0.032
3 standard reflections frequency: 120 min intensity decay: 1%

#### Refinement

*R*[*F* ^2^ > 2σ(*F* ^2^)] = 0.054
*wR*(*F* ^2^) = 0.188
*S* = 0.98
3643 reflections
256 parameters
1 restraint
H atoms treated by a mixture of independent and constrained refinement
Δρ_max_ = 0.19 e Å^−3^
Δρ_min_ = −0.20 e Å^−3^

### 

Data collection: *CAD-4 Software* (Enraf–Nonius, 1989 ▶); cell refinement: *CAD-4 Software*; data reduction: *XCAD4* (Harms & Wocadlo, 1995 ▶); program(s) used to solve structure: *SHELXS97* (Sheldrick, 2008 ▶); program(s) used to refine structure: *SHELXL97* (Sheldrick, 2008 ▶); molecular graphics: *ORTEP-3 for Windows* (Farrugia, 1997 ▶); software used to prepare material for publication: *SHELXTL* (Sheldrick, 2008 ▶).

## Supplementary Material

Crystal structure: contains datablocks global, I. DOI: 10.1107/S1600536808018746/hk2476sup1.cif

Structure factors: contains datablocks I. DOI: 10.1107/S1600536808018746/hk2476Isup2.hkl

Additional supplementary materials:  crystallographic information; 3D view; checkCIF report

## Figures and Tables

**Table 1:** Hydrogen-bond geometry (Å, °)

*D*—H⋯*A*	*D*—H	H⋯*A*	*D*⋯*A*	*D*—H⋯*A*
O1—H1*A*⋯O2	0.82	2.08	2.751 (5)	139
O2—H2*A*⋯O3^i^	0.85 (4)	2.11 (4)	2.906 (5)	156 (4)

Symmetry code: (i) 
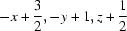
.

**Fig. 1.**
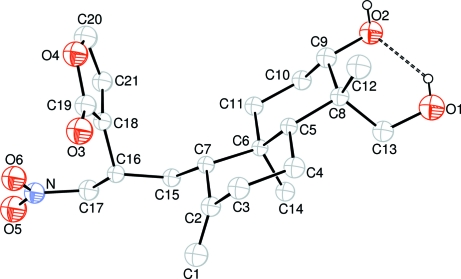
The molecular structure of the title molecule, with the atom-numbering scheme. Hydrogen bond is shown as dashed line.

**Fig. 2.**
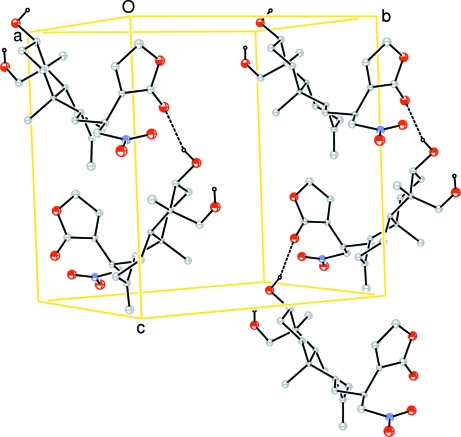
A partial packing diagram of (I). Hydrogen bonds are shown as dashed lines. H atoms not involved in hydrogen bonding have been omitted for clarity.

## supplementary crystallographic information

### Comment

Some derivatives of andrographolide are important chemical materials. We report herein the crystal structure of the title compound, (I).

In the molecule of (I), (Fig. 1), the bond lengths (Allen *et al.*, 1987) and angles are within normal ranges. Rings A (C2-C7) and B (C5/C6/C8-C11) adopt chair [φ = -86.32 (2)° and θ = 4.69 (3)° (for ring A) and φ = -148.49 (3)° and θ = 86.21 (3)° (for ring B)] conformations, having total puckering amplitudes, Q_T_, of 0.606 (3) Å and 0.642 (3) Å, respectively (Cremer & Pople, 1975). Ring C (O4/C18-C21) is, of course, planar. The intramolecular O-H···O hydrogen bond (Table 1) results in the formation of a six-membered non-planar ring: D (O1/H1A/O2/C8/C9/C13), in which it adopts twisted conformation, having total puckering amplitude, Q_T_, of 1.200 (3) Å (Cremer & Pople, 1975).

In the crystal structure, intermolecular O-H···O hydrogen bonds (Table 1) link the molecules into infinite chains along the c axis (Fig. 2), in which they may be effective in the stabilization of the structure.

### Experimental

For the preparation of the title compound, andrographolide (10 g) was dissolved in methanol (40 ml), and then nitromethane (16 ml), methanol (32 ml) and sodium methoxide (4.2 g) were added by stirring at room temperature. The reaction mixture was poured into ice salt water (120 ml). After the reaction finished, it was extracted with ethyl acetate, washed with saturated salt water and dryed with sodium sulfate. The product was filtrated and the organic layer was concentrated. Crystals suitable for X-ray analysis were obtained by slow evaporation of an ethyl acetate solution.

### Refinement

H2A atom was located in difference map and refined [O2-H2A = 0.843 (10) Å; U_iso_(H) = 0.080 Å^2^]. The remaining H atoms were positioned geometrically, with O-H = 0.82 Å (for OH) and C-H= 0.93 and 0.98 Å (for aromatic and methine H), 0.93 and 0.97 Å (for methylene H) and 0.96 Å (for methyl H), and constrained to ride on their parent atoms, with U_iso_(H) = xU_eq_(C,O), where x = 1.5 for OH and methyl H, and x = 1.2 for all other H atoms.

### Crystal data

**Table:**

C_21_H_31_NO_6_	*F*_000_ = 848
*M**_r_* = 393.47	*D*_x_ = 1.289 Mg m^−^^3^
Orthorhombic, *P*2_1_2_1_2_1_	Mo *K*α radiation λ = 0.71073 Å
Hall symbol: P 2ac 2ab	Cell parameters from 25 reflections
*a* = 11.503 (2) Å	θ = 10–13º
*b* = 13.151 (3) Å	µ = 0.09 mm^−^^1^
*c* = 13.434 (3) Å	*T* = 294 (2) K
*V* = 2032.2 (7) Å^3^	Block, colorless
*Z* = 4	0.40 × 0.20 × 0.20 mm

### Data collection

**Table:**

Enraf–Nonius CAD-4 diffractometer	*R*_int_ = 0.032
Radiation source: fine-focus sealed tube	θ_max_ = 25.2º
Monochromator: graphite	θ_min_ = 2.2º
*T* = 294(2) K	*h* = 0→13
ω/2θ scans	*k* = 0→15
Absorption correction: ψ scan(North *et al.*, 1968)	*l* = −16→16
*T*_min_ = 0.964, *T*_max_ = 0.982	3 standard reflections
3993 measured reflections	every 120 min
3643 independent reflections	intensity decay: 1%
2711 reflections with *I* > 2σ(*I*)	

### Refinement

**Table:**

Refinement on *F*^2^	Secondary atom site location: difference Fourier map
Least-squares matrix: full	Hydrogen site location: inferred from neighbouring sites
*R*[*F*^2^ > 2σ(*F*^2^)] = 0.055	H atoms treated by a mixture of independent and constrained refinement
*wR*(*F*^2^) = 0.188	*w* = 1/[σ^2^(*F*_o_^2^) + (0.1*P*)^2^ + 1.5*P*] where *P* = (*F*_o_^2^ + 2*F*_c_^2^)/3
*S* = 0.98	(Δ/σ)_max_ < 0.001
3643 reflections	Δρ_max_ = 0.19 e Å^−^^3^
256 parameters	Δρ_min_ = −0.20 e Å^−^^3^
1 restraint	Extinction correction: none
Primary atom site location: structure-invariant direct methods	

### Special details

**Table:**

Geometry. All e.s.d.'s (except the e.s.d. in the dihedral angle between two l.s. planes) are estimated using the full covariance matrix. The cell e.s.d.'s are taken into account individually in the estimation of e.s.d.'s in distances, angles and torsion angles; correlations between e.s.d.'s in cell parameters are only used when they are defined by crystal symmetry. An approximate (isotropic) treatment of cell e.s.d.'s is used for estimating e.s.d.'s involving l.s. planes.
Refinement. Refinement of F^2^ against ALL reflections. The weighted R-factor wR and goodness of fit S are based on F^2^, conventional R-factors R are based on F, with F set to zero for negative F^2^. The threshold expression of F^2^ > 2sigma(F^2^) is used only for calculating R-factors(gt) etc. and is not relevant to the choice of reflections for refinement. R-factors based on F^2^ are statistically about twice as large as those based on F, and R- factors based on ALL data will be even larger.

### Fractional atomic coordinates and isotropic or equivalent isotropic displacement parameters (Å^2^)

**Table:**

	*x*	*y*	*z*	*U*_iso_*/*U*_eq_	
O1	0.3022 (3)	0.2945 (3)	0.3635 (3)	0.0757 (11)	
H1A	0.3210	0.2868	0.4219	0.114*	
O2	0.4711 (3)	0.2805 (3)	0.5078 (2)	0.0664 (9)	
H2A	0.477 (5)	0.325 (3)	0.553 (3)	0.080*	
O3	0.9911 (4)	0.6195 (2)	0.1980 (3)	0.0767 (11)	
O4	1.0518 (4)	0.5831 (2)	0.3513 (2)	0.0705 (10)	
O5	1.2868 (3)	0.3264 (4)	0.0613 (3)	0.0923 (14)	
O6	1.2176 (3)	0.4675 (3)	0.1109 (3)	0.0743 (11)	
N	1.2092 (3)	0.3771 (4)	0.0954 (3)	0.0557 (10)	
C1	0.7547 (4)	0.4347 (4)	0.0088 (3)	0.0629 (13)	
H1B	0.7162	0.4713	−0.0404	0.075*	
H1C	0.8097	0.3861	−0.0088	0.075*	
C2	0.7313 (4)	0.4515 (3)	0.1031 (3)	0.0448 (10)	
C3	0.6437 (4)	0.5286 (3)	0.1353 (4)	0.0522 (11)	
H3A	0.6129	0.5636	0.0775	0.063*	
H3B	0.6806	0.5786	0.1780	0.063*	
C4	0.5440 (4)	0.4763 (3)	0.1922 (3)	0.0488 (11)	
H4A	0.4909	0.5274	0.2175	0.059*	
H4B	0.5011	0.4326	0.1470	0.059*	
C5	0.5913 (3)	0.4130 (3)	0.2788 (3)	0.0344 (8)	
H5A	0.6378	0.4615	0.3172	0.041*	
C6	0.6812 (3)	0.3322 (3)	0.2437 (3)	0.0331 (8)	
C7	0.7821 (3)	0.3923 (3)	0.1901 (3)	0.0359 (8)	
H7A	0.8088	0.4433	0.2380	0.043*	
C8	0.4951 (3)	0.3781 (3)	0.3532 (3)	0.0407 (9)	
C9	0.5559 (4)	0.3237 (4)	0.4396 (3)	0.0500 (10)	
H9A	0.6021	0.3740	0.4761	0.060*	
C10	0.6359 (4)	0.2395 (3)	0.4063 (3)	0.0496 (11)	
H10A	0.5908	0.1876	0.3725	0.059*	
H10B	0.6717	0.2085	0.4642	0.059*	
C11	0.7308 (3)	0.2784 (3)	0.3364 (3)	0.0445 (10)	
H11A	0.7786	0.2216	0.3153	0.053*	
H11B	0.7802	0.3255	0.3724	0.053*	
C12	0.4332 (5)	0.4722 (4)	0.3948 (4)	0.0646 (13)	
H12A	0.3736	0.4513	0.4405	0.097*	
H12B	0.3988	0.5098	0.3411	0.097*	
H12C	0.4884	0.5144	0.4289	0.097*	
C13	0.4032 (3)	0.3085 (4)	0.3047 (4)	0.0519 (11)	
H13A	0.3806	0.3375	0.2412	0.062*	
H13B	0.4380	0.2427	0.2917	0.062*	
C14	0.6321 (4)	0.2524 (3)	0.1714 (3)	0.0465 (10)	
H14A	0.5702	0.2157	0.2032	0.070*	
H14B	0.6925	0.2058	0.1527	0.070*	
H14C	0.6027	0.2857	0.1130	0.070*	
C15	0.8890 (3)	0.3280 (3)	0.1645 (3)	0.0422 (9)	
H15A	0.8742	0.2914	0.1031	0.051*	
H15B	0.9011	0.2783	0.2167	0.051*	
C16	1.0006 (3)	0.3920 (3)	0.1522 (3)	0.0392 (9)	
H16A	0.9876	0.4425	0.0998	0.047*	
C17	1.0987 (4)	0.3222 (4)	0.1201 (4)	0.0555 (12)	
H17A	1.1141	0.2739	0.1731	0.067*	
H17B	1.0738	0.2839	0.0621	0.067*	
C18	1.0289 (3)	0.4467 (3)	0.2473 (3)	0.0400 (9)	
C19	1.0200 (4)	0.5570 (4)	0.2574 (3)	0.0534 (11)	
C20	1.0817 (5)	0.4937 (4)	0.4047 (4)	0.0668 (13)	
H20A	1.0320	0.4855	0.4625	0.080*	
H20B	1.1620	0.4965	0.4268	0.080*	
C21	1.0646 (4)	0.4099 (4)	0.3342 (3)	0.0523 (11)	
H21A	1.0769	0.3415	0.3481	0.063*	

### Atomic displacement parameters (Å^2^)

**Table:**

	*U*^11^	*U*^22^	*U*^33^	*U*^12^	*U*^13^	*U*^23^
O1	0.0430 (18)	0.083 (2)	0.101 (3)	−0.0086 (17)	0.0210 (19)	0.002 (2)
O2	0.069 (2)	0.081 (2)	0.0489 (18)	−0.007 (2)	0.0189 (17)	0.0045 (16)
O3	0.112 (3)	0.0437 (17)	0.075 (2)	0.001 (2)	−0.020 (2)	0.0099 (17)
O4	0.094 (3)	0.061 (2)	0.0572 (19)	−0.0127 (19)	−0.0167 (19)	−0.0096 (17)
O5	0.055 (2)	0.131 (4)	0.091 (3)	0.000 (2)	0.018 (2)	−0.038 (3)
O6	0.050 (2)	0.078 (3)	0.095 (3)	−0.0172 (18)	0.0090 (19)	0.015 (2)
N	0.038 (2)	0.089 (3)	0.0404 (19)	0.000 (2)	0.0067 (16)	−0.003 (2)
C1	0.054 (3)	0.080 (3)	0.055 (3)	−0.012 (3)	0.001 (2)	0.014 (3)
C2	0.041 (2)	0.047 (2)	0.047 (2)	−0.0133 (19)	0.0018 (18)	0.0099 (19)
C3	0.052 (3)	0.044 (2)	0.061 (3)	0.002 (2)	−0.005 (2)	0.015 (2)
C4	0.044 (2)	0.048 (2)	0.055 (3)	0.0084 (19)	−0.001 (2)	0.016 (2)
C5	0.0328 (19)	0.0331 (19)	0.0373 (19)	−0.0008 (15)	−0.0054 (15)	−0.0029 (15)
C6	0.0341 (19)	0.0299 (18)	0.0353 (19)	−0.0043 (16)	−0.0006 (16)	−0.0001 (16)
C7	0.0347 (19)	0.0321 (18)	0.041 (2)	−0.0026 (17)	−0.0040 (17)	−0.0023 (16)
C8	0.037 (2)	0.0405 (19)	0.045 (2)	0.0007 (18)	0.0087 (18)	−0.0034 (17)
C9	0.054 (2)	0.058 (3)	0.038 (2)	−0.012 (2)	0.0076 (19)	−0.0002 (19)
C10	0.051 (3)	0.056 (2)	0.042 (2)	0.004 (2)	−0.004 (2)	0.018 (2)
C11	0.041 (2)	0.043 (2)	0.049 (2)	0.0014 (18)	0.0008 (19)	0.0079 (18)
C12	0.064 (3)	0.058 (3)	0.072 (3)	0.006 (2)	0.017 (3)	−0.006 (2)
C13	0.036 (2)	0.056 (3)	0.064 (3)	−0.003 (2)	0.004 (2)	0.008 (2)
C14	0.051 (2)	0.039 (2)	0.049 (2)	−0.0112 (19)	0.007 (2)	−0.0069 (19)
C15	0.040 (2)	0.0364 (19)	0.050 (2)	−0.0072 (17)	0.0065 (18)	−0.0022 (17)
C16	0.038 (2)	0.0405 (19)	0.039 (2)	−0.0032 (18)	0.0041 (17)	0.0026 (17)
C17	0.041 (2)	0.065 (3)	0.061 (3)	−0.005 (2)	0.011 (2)	−0.009 (2)
C18	0.033 (2)	0.044 (2)	0.043 (2)	−0.0048 (17)	−0.0007 (18)	0.0054 (18)
C19	0.058 (3)	0.049 (2)	0.053 (2)	−0.008 (2)	−0.005 (2)	−0.001 (2)
C20	0.065 (3)	0.087 (4)	0.048 (3)	−0.009 (3)	−0.007 (2)	0.004 (3)
C21	0.048 (2)	0.058 (3)	0.050 (2)	−0.001 (2)	−0.004 (2)	0.011 (2)

### Geometric parameters (Å, °)

**Table:**

O1—C13	1.417 (5)	C8—C13	1.542 (6)
O1—H1A	0.8200	C9—C10	1.509 (6)
O2—C9	1.453 (5)	C9—H9A	0.9800
O2—H2A	0.85 (4)	C10—C11	1.528 (6)
O3—C19	1.194 (5)	C10—H10A	0.9700
C3—C4	1.540 (6)	C10—H10B	0.9700
C3—H3A	0.9700	C11—H11A	0.9700
C3—H3B	0.9700	C11—H11B	0.9700
O4—C19	1.358 (5)	C12—H12A	0.9600
O4—C20	1.420 (6)	C12—H12B	0.9600
N—O5	1.205 (5)	C12—H12C	0.9600
N—O6	1.211 (5)	C13—H13A	0.9700
N—C17	1.499 (6)	C13—H13B	0.9700
C1—C2	1.314 (6)	C14—H14A	0.9600
C1—H1B	0.9300	C14—H14B	0.9600
C1—H1C	0.9300	C14—H14C	0.9600
C2—C3	1.494 (6)	C15—C16	1.544 (5)
C2—C7	1.521 (6)	C15—H15A	0.9700
C4—C5	1.530 (5)	C15—H15B	0.9700
C4—H4A	0.9700	C16—C18	1.502 (6)
C4—H4B	0.9700	C16—C17	1.517 (6)
C5—C6	1.556 (5)	C16—H16A	0.9800
C5—C8	1.561 (5)	C17—H17A	0.9700
C5—H5A	0.9800	C17—H17B	0.9700
C6—C14	1.538 (5)	C18—C21	1.329 (6)
C6—C11	1.542 (5)	C18—C19	1.460 (6)
C6—C7	1.578 (5)	C20—C21	1.467 (7)
C7—C15	1.531 (5)	C20—H20A	0.9700
C7—H7A	0.9800	C20—H20B	0.9700
C8—C9	1.532 (6)	C21—H21A	0.9300
C8—C12	1.533 (6)		
			
C13—O1—H1A	109.5	H10A—C10—H10B	107.9
O5—N—O6	123.3 (4)	C10—C11—C6	112.7 (3)
O5—N—C17	116.5 (4)	C10—C11—H11A	109.1
O6—N—C17	120.2 (4)	C6—C11—H11A	109.1
C2—C1—H1B	120.0	C10—C11—H11B	109.1
C2—C1—H1C	120.0	C6—C11—H11B	109.1
H1B—C1—H1C	120.0	H11A—C11—H11B	107.8
C1—C2—C3	122.1 (4)	C8—C12—H12A	109.5
C1—C2—C7	125.2 (4)	C8—C12—H12B	109.5
C3—C2—C7	112.6 (3)	H12A—C12—H12B	109.5
C9—O2—H2A	97 (4)	C8—C12—H12C	109.5
C2—C3—C4	110.1 (3)	H12A—C12—H12C	109.5
C2—C3—H3A	109.6	H12B—C12—H12C	109.5
C4—C3—H3A	109.6	O1—C13—C8	113.8 (4)
C2—C3—H3B	109.6	O1—C13—H13A	108.8
C4—C3—H3B	109.7	C8—C13—H13A	108.8
H3A—C3—H3B	108.2	O1—C13—H13B	108.8
C19—O4—C20	109.0 (4)	C8—C13—H13B	108.8
C5—C4—C3	110.8 (3)	H13A—C13—H13B	107.7
C5—C4—H4A	109.5	C6—C14—H14A	109.5
C3—C4—H4A	109.5	C6—C14—H14B	109.5
C5—C4—H4B	109.5	H14A—C14—H14B	109.5
C3—C4—H4B	109.5	C6—C14—H14C	109.5
H4A—C4—H4B	108.1	H14A—C14—H14C	109.5
C4—C5—C6	112.2 (3)	H14B—C14—H14C	109.5
C4—C5—C8	113.3 (3)	C7—C15—C16	113.1 (3)
C6—C5—C8	117.7 (3)	C7—C15—H15A	109.0
C4—C5—H5A	103.9	C16—C15—H15A	109.0
C6—C5—H5A	103.9	C7—C15—H15B	109.0
C8—C5—H5A	103.9	C16—C15—H15B	109.0
C14—C6—C11	109.5 (3)	H15A—C15—H15B	107.8
C14—C6—C5	114.4 (3)	C18—C16—C17	111.8 (4)
C11—C6—C5	108.3 (3)	C18—C16—C15	110.6 (3)
C14—C6—C7	108.9 (3)	C17—C16—C15	108.6 (3)
C11—C6—C7	109.0 (3)	C18—C16—H16A	108.6
C5—C6—C7	106.6 (3)	C17—C16—H16A	108.6
C2—C7—C15	114.7 (3)	C15—C16—H16A	108.6
C2—C7—C6	108.9 (3)	N—C17—C16	113.7 (4)
C15—C7—C6	114.7 (3)	N—C17—H17A	108.8
C2—C7—H7A	105.9	C16—C17—H17A	108.8
C15—C7—H7A	105.9	N—C17—H17B	108.8
C6—C7—H7A	105.9	C16—C17—H17B	108.8
C9—C8—C12	108.2 (4)	H17A—C17—H17B	107.7
C9—C8—C13	110.9 (3)	C21—C18—C19	107.6 (4)
C12—C8—C13	108.3 (4)	C21—C18—C16	129.7 (4)
C9—C8—C5	107.4 (3)	C19—C18—C16	122.7 (4)
C12—C8—C5	109.0 (3)	O3—C19—O4	121.5 (4)
C13—C8—C5	112.9 (3)	O3—C19—C18	129.9 (4)
O2—C9—C10	108.0 (4)	O4—C19—C18	108.6 (4)
O2—C9—C8	110.7 (4)	O4—C20—C21	105.2 (4)
C10—C9—C8	113.3 (3)	O4—C20—H20A	110.7
O2—C9—H9A	108.2	C21—C20—H20A	110.7
C10—C9—H9A	108.2	O4—C20—H20B	110.7
C8—C9—H9A	108.2	C21—C20—H20B	110.7
C9—C10—C11	111.9 (3)	H20A—C20—H20B	108.8
C9—C10—H10A	109.2	C18—C21—C20	109.6 (4)
C11—C10—H10A	109.2	C18—C21—H21A	125.2
C9—C10—H10B	109.2	C20—C21—H21A	125.2
C11—C10—H10B	109.2		
			
C1—C2—C3—C4	117.9 (5)	C5—C8—C9—C10	−53.2 (4)
C7—C2—C3—C4	−58.4 (5)	O2—C9—C10—C11	−178.3 (3)
C2—C3—C4—C5	54.5 (5)	C8—C9—C10—C11	58.7 (5)
C3—C4—C5—C6	−56.8 (4)	C9—C10—C11—C6	−57.0 (5)
C3—C4—C5—C8	167.0 (3)	C14—C6—C11—C10	−74.5 (4)
C4—C5—C6—C14	−62.0 (4)	C5—C6—C11—C10	50.8 (4)
C8—C5—C6—C14	72.1 (4)	C7—C6—C11—C10	166.4 (3)
C4—C5—C6—C11	175.6 (3)	C9—C8—C13—O1	72.8 (4)
C8—C5—C6—C11	−50.3 (4)	C12—C8—C13—O1	−45.8 (5)
C4—C5—C6—C7	58.4 (4)	C5—C8—C13—O1	−166.6 (4)
C8—C5—C6—C7	−167.5 (3)	C2—C7—C15—C16	75.8 (4)
C1—C2—C7—C15	15.6 (6)	C6—C7—C15—C16	−157.0 (3)
C3—C2—C7—C15	−168.2 (3)	C7—C15—C16—C18	60.9 (4)
C1—C2—C7—C6	−114.4 (5)	C7—C15—C16—C17	−176.2 (4)
C3—C2—C7—C6	61.8 (4)	O5—N—C17—C16	−173.8 (4)
C14—C6—C7—C2	64.7 (4)	O6—N—C17—C16	6.9 (6)
C11—C6—C7—C2	−175.9 (3)	C18—C16—C17—N	−63.8 (5)
C5—C6—C7—C2	−59.2 (4)	C15—C16—C17—N	174.0 (4)
C14—C6—C7—C15	−65.3 (4)	C17—C16—C18—C21	−52.3 (6)
C11—C6—C7—C15	54.1 (4)	C15—C16—C18—C21	68.9 (5)
C5—C6—C7—C15	170.8 (3)	C17—C16—C18—C19	127.8 (4)
C4—C5—C8—C9	−175.4 (3)	C15—C16—C18—C19	−111.0 (4)
C6—C5—C8—C9	51.0 (4)	C20—O4—C19—O3	−179.9 (5)
C4—C5—C8—C12	−58.4 (5)	C20—O4—C19—C18	−0.1 (5)
C6—C5—C8—C12	168.0 (4)	C21—C18—C19—O3	−179.8 (5)
C4—C5—C8—C13	62.1 (4)	C16—C18—C19—O3	0.1 (8)
C6—C5—C8—C13	−71.5 (4)	C21—C18—C19—O4	0.4 (5)
C12—C8—C9—O2	67.8 (4)	C16—C18—C19—O4	−179.7 (3)
C13—C8—C9—O2	−50.9 (4)	C19—O4—C20—C21	−0.2 (5)
C5—C8—C9—O2	−174.7 (3)	C19—C18—C21—C20	−0.5 (5)
C12—C8—C9—C10	−170.7 (4)	C16—C18—C21—C20	179.6 (4)
C13—C8—C9—C10	70.6 (4)	O4—C20—C21—C18	0.5 (5)

### Hydrogen-bond geometry (Å, °)

**Table:**

*D*—H···*A*	*D*—H	H···*A*	*D*···*A*	*D*—H···*A*
O1—H1A···O2	0.82	2.08	2.751 (5)	139
O2—H2A···O3^i^	0.85 (4)	2.11 (4)	2.906 (5)	156 (4)

Symmetry codes: (i) −*x*+3/2, −*y*+1, *z*+1/2.
